# Construction and validity of educational technology in audiovisual media on premature newborn care

**DOI:** 10.1590/0034-7167-2022-0403

**Published:** 2023-11-10

**Authors:** Marcelo Victor Freitas Nascimento, Willyane de Andrade Alvarenga, Marcia Teles de Oliveira Gouveia, Herla Maria Furtado Jorge, Marianne Rocha Duarte de Carvalho, Jackeline Vieira Amaral, Silvana Santiago da Rocha

**Affiliations:** I Universidade Federal do Piauí. Teresina, Piauí, Brazil; II Empresa Brazileira de Serviços Hospitalares. Brasília, Distrito Federal, Brazil; III Universidade de São Paulo. Ribeirão Preto, São Paulo, Brazil

**Keywords:** Premature Birth, Educational Technology, Video-Audio Media, Neonatal Nursing, Health Education, Recién Nacido Prematuro, Tecnología Educacional, Medios Audiovisuales, Enfermería Neonatal, Educación en Salud, Recém-Nascido Prematuro, Tecnologia Educacional, Mídia Audiovisual, Enfermagem Neonatal, Educação em Saúde

## Abstract

**Objectives:**

to construct and validate an educational video storyboard about care for premature newborns at home.

**Methods:**

a methodological study, with the construction of an educational video storyboard, validated with 14 judges. Content was selected from scoping review. For data collection, a validated instrument was used. The criterion for validity was agreement greater than 80%, analyzed using the Content Validity Index.

**Results:**

the storyboard construction was guided by the Cognitive Theory of Multimedia Learning theoretical framework. Construction and validity took place from May to December 2020. The storyboard’s final version lasted 10 minutes, and was validated in terms of objective, structure, presentation and relevance, with a Content Validity Index of 0.9.

**Conclusions:**

the storyboard of the educational video proved to be valid and adequate for health promotion in developing care for premature newborns at home.

## RESUMO

**Objetivos:**

construir e validar *storyboard* de vídeo educativo acerca dos cuidados com recém-nascido prematuro no domicílio.

**Métodos:**

estudo metodológico, com construção de *storyboard* de vídeo educativo, validado com 14 juízes. Selecionou-se o conteúdo a partir de revisão de escopo. Para coleta dos dados, utilizou-se instrumento validado. O critério para validação foi concordância superior a 80%, analisada por meio do Índice de Validação de Conteúdo.

**Resultados:**

a construção do *storyboard* foi guiada pelo referencial teórico da Teoria Cognitiva da Aprendizagem Multimídia. A construção e a validação ocorreram nos meses de maio a dezembro de 2020. A versão final do *storyboard* contém duração de 10 minutos, e foi validada quanto aos objetivos, estrutura, apresentação e relevância, com Índice de Validade de Conteúdo de 0,9.

**Conclusões:**

o *storyboard* do vídeo educativo se mostrou válido e adequado para promoção da saúde no desenvolvimento de cuidados ao recém-nascido prematuro no domicílio.

## RESUMEN

**Objetivos:**

construir y validar un video storyboard educativo sobre el cuidado del recién nacido prematuro en el domicilio.

**Métodos:**

estudio metodológico, con la construcción de un storyboard de video educativo, validado con 14 jueces. El contenido fue seleccionado de la revisión del alcance. Para la recolección de datos se utilizó un instrumento validado. El criterio de validación fue concordancia superior al 80%, analizado mediante el Índice de Validación de Contenido.

**Resultados:**

la construcción del storyboard fue guiada por el referencial teórico de la Teoría Cognitiva del Aprendizaje Multimedia. La construcción y validación se llevó a cabo de mayo a diciembre de 2020. La versión final del storyboard tiene una duración de 10 minutos y fue validada en cuanto a objetivos, estructura, presentación y relevancia, con un Índice de Validez de Contenido de 0,9.

**Conclusiones:**

el storyboard del video educativo se mostró válido y adecuado para la promoción de la salud en el desarrollo de la atención al recién nacido prematuro en el domicilio.

## INTRODUCTION

The birth of a newborn (NB) before 37 weeks is a global public health problem, as it is the main risk factor for neonatal mortality, and the second cause, in children up to 5 years old, associated with long hospitalization duration^(1)^. On average, 15 million babies are born prematurely in the world^(2)^. With a rate of 42%, Brazil has a higher recurrent rate than those reported in studies carried out in other countries, such as the Netherlands (29.3%), Japan (22.3%) and the United States (21%)^(3)^. Prematurity occurs in more than one in ten births^(1)^, due to the persistence of preconception problems or during pregnancy^(4)^.

Taking into account that the main cause of hospitalizations in neonatal units is prematurity^(5)^, premature newborns (PNB) are at high risk of health complications in the short and long term^(3)^. The risk of complications and coordination of care makes the moment of hospital discharge crucial for offering instructions directed at PNB’s needs at home^(5)^. Studies show that the role of parents, family members and/or caregivers at home, lack of preparation for parenthood, lower levels of education, low socioeconomic status, hospitalization and experiencing ambivalent feelings, together with pain and isolation, contribute to a difficult emotional situation regarding the experience of home care^(4, 6)^.

The recurrence of prematurity is influenced by the effects of organization of obstetric care. In this way, worldwide, commitments to improve health care for pregnant women and NB have increased^(^3,7^)^. Standards for qualified care in labor and childbirth have been established in Brazil, such as the Comprehensive Care Program for Women’s Health (PAISM - *Programa de Assistência Integral à Saúde da Mulher*), evidence-based clinical guidelines for normal delivery care^(8)^, the Prenatal Care and Birth Humanization Program and the Pact for Maternal and Neonatal Mortality Reduction, which culminated in the advent of Stork Network (RC)^(7, 8)^.

Considering the high rates of prematurity, the complexity of care transition reveals the responsibilities of nursing professionals in the support, guidance and instrumentalization of parents and caregivers during hospitalization for home care^(9)^. The construction and implementation of educational materials can be complementary instruments and facilitators of the teaching-learning process, allowing the construction of knowledge through involvement, participation and exchange of experiences^(10, 11)^.

Among the various information, guidance and communication technologies, there is technology that uses an audiovisual resource in the format of an educational video. Technologies in this format have assumed a proposal of sophistication in the teaching-learning relationship^(12)^, since, through it, it is possible to catch the public’s attention and interaction as well as to arouse their curiosity in relation to the themes addressed^(9, 12, 13)^. The educational video uses an education process through mechanisms linked to audio and images^(14)^. In view of this, it is a potential tool for health education and nursing care, which shows the relevance of studies on the construction and validity of this tool, whether for teaching nursing or for educating the target audience. An example of this type of study is the one that elaborated and validated a video with an animated cartoon about the home bath of full-term NB directed to family members and caregivers^(15)^.

## OBJECTIVES

To construct and validate an educational video storyboard about PNB care at home.

## METHODS

### Ethical aspects

The research complied with Resolution 466/2012 principles, being approved by the Research Ethics Committee of the *Universidade Federal do Piauí*.

### Study design, place and study

This is methodological research carried out from May to December 2020, in the municipality of Teresina, Piauí. Data collection for storyboard content validity was carried out online, that allowed building and validating a technological strategy that can be implemented both in educational and care environments^(5, 9, 12)^. The storyboard construction followed the 12 principles (coherence, signaling, redundancy, spatial contiguity, temporal contiguity, segmentation, pre-training, modality, multimedia, personalization, voice and image) of the Cognitive Theory of Multimedia Learning (CTML)^(16)^ theoretical framework as well as the 3 stages (pre-production, production and post-production) of Kindem’s and Musburger’s methodological framework^(17)^. Due to the fact that it is not possible for the visual and sound elements that make up a video to be presented only in the script, it was decided to carry out the storyboard validity process, as in other nursing studies^(18, 19, 20)^. The SQUIRE 2.0 instrument from the Equator network was used to guide the methodology.

### Sample: eligibility criteria for professionals to compose the judge committee

Content validity was carried out by experts with experience in the subject of this study, according to the score (maximum of 11), based on pre-established criteria^(21)^. Professionals who obtained at least five points were included (graduate degree, participation in a project, work or publication in the area of child health, prematurity, neonatology, obstetrics, construction and validity of instruments, experience in video development and time working at least one year in the area). The choice to include only nurses was due to the importance of nursing in the development and use of technologies for health education. Nurses who sent incomplete forms and after the established deadline were excluded.

Expert identification was carried out by searching the curricula available on the *Plataforma Lattes* of the Brazilian National Council for Scientific and Technological Development (*Conselho Nacional de Desenvolvimento Científico e Tecnológico*). Invitations were sent to 35 experts to participate in the research, via e-mail and/or via a multiplatform instant messaging application (WhatsApp®), who met the established criteria^(21)^, through intentional sampling^(22)^. After acceptance, the access link to the questionnaire was sent by email. The final sample resulted in 14 experts.

### Study protocol

The study was developed in three stages and six distinct phases^(17)^. The first stage, pre-production, consisted of two phases, such as scoping review, synopsis construction, argument and script. Identifying parents’ and family members’ needs regarding PNB care at home and listing the key elements for building the audiovisual media script occurred through a scope review, being structured according to the Activities of Daily Living Model thematic axes^(23, 24, 25)^.

The included studies were obtained through the PCC search strategy (P: Population, C: Concept and C: Context) and the following research question: what care is provided to the BNP by caregivers at home? The searches and selection of studies were carried out by two reviewers who were doctoral students in nursing at the *Universidade Federal do Piauí*, in the PubMed/MEDLINE, Cumulative Index to Nursing and Allied Health Literature (CINAHL), Web of Science and Scopus databases. Descriptors were used, Caregivers; Infant, Premature; Infant, Extremely Premature; Housing, Family Patient; Lodging, and their corresponding keywords, combined with Boolean operators AND and OR^(25)^.

Seeking to expand the recommendations of PNB care, consultation was carried out with books, texts on nursing, neonatology, neonatal intensive care and manuals from the Ministry of Health in the field of neonatology. The second phase was based on script construction, according to the CTML’s 12 principles^(16)^, organized by sequence: title presentation, synopsis and argument as well as description of action, animation and voice-over^(17)^.

The production stage had three phases, image development, animations and storyboard, ending the stage with storyboard validity by experts. In the first phase, there was the elaboration of images by a nurse with expertise in the area of construction of images for educational actions. The second was carried out by a design company, which consisted of structuring the storyboard in frames with three columns (audio/narration, images/scenes and photos), arranging the script content in the audio/narration column, detailing of texts and actions for recording, in the image/scene column and the images, logos and animations, in the photo column. The third phase consisted of validity by selected nursing professionals, according to previously established scores and criteria^(21)^.

For the validity phase, experts received a link to access the electronic form, built using an online forms application (Google Forms), which contained the letter of invitation to participate in the expert committee, the Informed Consent Form, the video storyboard, a characterization form for experts and the Health Education Content Validity Instrument (IVCES - *Instrumento de Validação de Conteúdo Educativo em Saúde*)^(26)^, which contained 18 questions, divided into three domains (objectives, structure/presentation and relevance). Judges assessed content using a Likert-type scale, with 3 points being assigned to “adequate without significant considerations”, 2 to “adequate with suggestions”, 1 to “partially adequate” and 0 to “inadequate”. The form also had space for recording suggestions or justifications from expert judges for each IVCES domain^(26)^.

The last step, consisting of one phase, editing, consisted of periodic meetings with the technical team, under the guidance of the researchers, totaling 20 hours dedicated to the corrections requested by experts. There were changes in some images as well as in the text of some animations in the video.

Images were designed with the help of the Autodesk SketchBook graphics software application, which allows creating drawings using graphic resources and layer division. Animations were performed using Adobe’s Premiere tool for projecting images, and were animated using the Adobe After Effects CS6 program.

In the post-production stage, the editing, finalization and final organization of the storyboard of the educational video were carried out. The storyboard has 44 screens, which were created in 500dpi resolution and saved in jpeg format.

### Analysis of results, and statistics

The data was organized using Microsoft Excel® 2021. Item adequacy was analyzed using the Content Validity Index (CVI), a method used in the health area^(27)^. The item was considered valid if the proportion of experts’ agreement was equal to or greater than 0.80^(28, 29)^. The data generated were analyzed using the Statistical Package for the Social Sciences (SPSS) program, version 22.0, for Windows, and grouped into tables, allowing data interpretation and descriptive quantitative explanation.

## RESULTS

The final sample of the scoping review, omitted for peer review, consisted of 18 articles. The studies were developed by researchers from Brazil (n=10), India (n=2), Iran (n=1), Bangladesh (n=1), United States (n=1), Malawi (n=1), Rwanda (n=1) and Colombia (n=1). Five of the studies were published in 2017 and three in 2021. The years 2020, 2013 and 2007 had 2 studies each, and 4 in the years 2006, 2009, 2010 and 2018, respectively. Regarding the methodological design used, twelve were descriptive studies, two used Grounded Theory, two were randomized clinical trials, one was an ethnographic study and another was a phenomenological study.

From the scoping review, caregivers’ needs were identified when performing home care with BNP. Thus, there was a demand for information, support, security and autonomy for bathing, cleaning, changing diapers, feeding, infection prevention, skin care, temperature maintenance, crying, sleep and rest. Absence and difficulties also emerged for: food supply (breast milk and/or infant formula); preparing food and expressing breast milk directly from the breasts; maintaining temperature and practicing the kangaroo position; bath, hygiene and diaper change; sleep and rest; insecurity and care in the face of intercurrences (apnea, reflux, bradycardia, milk engorgement); medication administration; need for social, economic support; and need for follow-up by the health team after hospital discharge.

**Figure 1:**
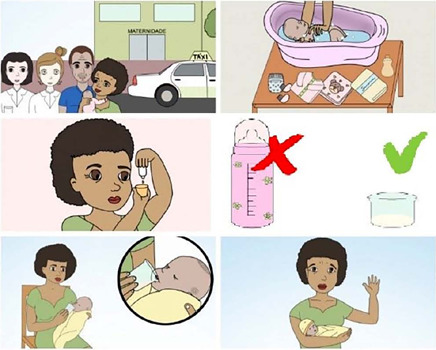
Scenes from the storyboard “O cuidado do prematuro”, Teresina, Piauí, Brazil, 2021

The construction and validity of the educational video’s storyboard were developed from May to December 2020. The final version of the storyboard entitled “*O cuidado do prematuro*” (Premature infant care) lasts 10 minutes, which can be seen in front of some scenes in Figure 1.

Storyboard content validity was carried out by 14 nurses from the areas of child health and health technology validity. The nurses who participated in content validity met at least three requirements for the selection of expert judges^(21)^. The average age of judges was 32.8 (±4.5) years, ranging from 28 to 45 years. Female professionals predominated (10; 71.4%), working predominantly in teaching (7; 50.0%) or also in care (6; 42.9%), with an average training time (9; ±4.3) years, ranging from 4 to 20 years. The majority had a link with a public higher education institution (8; 57.1%) or with a maternity hospital (4; 28.6%), concentrated in the states of Piauí (8; 57.1%). and Pernambuco (3; 21.4%). They had a master’s degree (10; 71.4%) or a doctoral degree (4; 28.6%), with scientific publications in child health and/or prematurity (11; 78.6%). All of them participated in some type of research/extension groups/projects within the last five years. Among them, ten (71.4%) reported having experience in child health for 4.5 (±2.7) years, on average (minimum of one year and maximum of ten years).

Of the 18 items judged by experts, the CVI of the criteria referring to educational content ranged from 85.7% (structure and presentation) to 100.0% (relevance). As for objectives (CVI=92.9%), the lowest index referred to adequacy to the teaching-learning process, with 78.6%, being modified according to experts’ suggestions.

In terms of structure and presentation, even though some of the items were judged as adequate or partially adequate, modifications and suggestions were accepted, especially when it came to the items of language appropriate to the target audience, language appropriate to the educational material and logical sequence of ideas. All relevant items had a maximum CVI (100.0%), as shown in Table 1.

**Table 1:** Content Validity Index of the Health Educational Content Validity Instrument criteria, as assessed by judges, Teresina, Piauí, Brazil, 2021

Criterion	CVI
Objectives	0.929
	Considers the proposed topic
1.000	
Adequate for the teaching-learning process	0.786
	Clarifies doubts about the topic addressed
0.929	
Provides reflection on the topic	0.929
Encourages behavior change	1.000
Structure and presentation	0.857
Language adequate for the target audience	0.643
Appropriate language for educational material	0.714
Interactive language, allowing active involvement in the educational process	0.929
Correct information	1.000
Objective information	0.929
Enlightening information	1.000
Necessary information	0.857
Logical sequence of ideas	0.714
Current topic	0.929
Appropriate text size	0.857
Relevance	1.000
Encourages learning	1.000
Contributes to knowledge in the area	1.000
Arouses interest in the topic	1.000
CVI* total	0.901

Elaborated from the Health Educational Content Validity Instrument^(25)^ items; *CVI - Content Validity Index.

Thus, as all domains with agreement among judges were greater than 80%, the content of “*O cuidado do prematuro*” was valid in terms of objectives, structure, presentation and relevance. Chart 1 shows the modifications, based on judges’ suggestions, with the aim of improving item content and understanding.

**Chart 1:** Modifications made to the educational video’s storyboard, according to judges’ assessment, Teresina, Piauí, Brazil, 2021

Assessed requirement	Before expert review	After expert review
Language adequate for the target audience	*“Quais os procedimentos devem ser tomados?”* (What procedures must be taken?)	*“Quais as etapas para alimentar ele corretamente?”* (What are the steps to feed them correctly??)
Language adequate for the target audience	*“...diminui o índice de desmame precoce.”* (…decreases the rate of early weaning)	*“... e faz o bebê se alimentar com o leite do peito por mais tempo.”* (…and makes babies breastfeed longer)
Logical sequence and appropriate language for the target audience	*“A frequência em oferecer o leite ao prematuro vai depender de quantas vezes ele solicita o peito.”* (The frequency of offering milk to premature babies will depend on how often they request the breast)	*“A quantidade de vezes que você deve dar o leite ao bebê prematuro vai depender de quantas vezes ele parece querer o peito.”* (The amount of times you should give premature babies breast milk will depend on how often they seem to want the breast)
Structure and presentation	*“Ofereça primeiro ao bebê que mame direto do peito.”* (First offer babies to feed straight from the breast)	*“Primeiro tente que o bebê mame no peito. Se o bebê não quiser ou não conseguir mamar no peito, será necessário dar o leite no copinho, para isso, coloque-o na posição semissentada.”* (First try to get babies to breastfeed. If babies do not want or are unable to breastfeed, it will be necessary to give the milk in a cup, for this, place it in the semi-sitting position)
Language adequate for the target audience	*“... no lábio superior do bebê para evitar que empurre o copo para fora.”* (…on babies’ upper lip to prevent pushing the cup out)	*“... no lábio de cima do bebê, para que ele não empurre o copo com a língua.”* (…on babies’ upper lip, so they don’t push the cup with their tongue)
Language adequate for the target audience	*“... espere que o bebê absorva o leite, e não force obrigando-o a engolir.”* (…wait for the baby to absorb the milk, and do not force it to swallow)	*“... espere que o leite escorra para dentro da boca do bebê e que ele engula no tempo dele, não force o bebê a engolir derramando grande quantidade de leite na sua boca.”* (…wait for the milk to flow into the babies’ mouth and swallow in their time, do not force babies to swallow by pouring large amounts of milk into their mouth)
Logical sequence and appropriate language for the target audience	“Antes de colocá-lo no berço, *espere* ele arrotar, após isso deite o do seu lado direito.” (Before putting them in the crib, wait for them to burp, then lay them on your right side)	*“Antes de colocá-lo no berço, espere ele arrotar, para isso, acomode ele no seu colo, com a cabeça para cima e movimente-o o mínimo possível para que ele não vomite.”* (Before putting them in the crib, wait for them to burp, for that, place them on your lap, with their heads up and move them as little as possible so that they don’t vomit)
Language adequate for the target audience	*“... após isso deite-o do seu lado direito por.”* (…after that, lay them on their right side for)	*“... depois que o bebê arrotar, deite ele de lado no berço, com o lado direito em contato com o colchão.”* (after babies burp, lay them on their side in the crib, right side in contact with the mattress)
Language adequate for the target audience	*“É válido lembrar que a ordenha mamária é no mínimo 6 vezes ao dia...”* (It is worth remembering that breast milking is at least 6 times a day)	*“Se seu bebê se alimentar pelo copinho, você precisará tirar leite do seu peito, pelo menos 6 vezes por dia...”* (If your baby is cup-feeding, you will need to express milk from your breast at least 6 times a day)
Logical sequence and appropriate language for the target audience	*“... em que se deve começar lavando as mãos, em seguida realizar massagens suaves no peito para estimular a decida do leite com movimentos em círculos e depois de cima para baixo.”* (…in which you should start by washing your hands, then gently massage the breast to stimulate the milk to come out with circular movements and then from top to bottom)	*“... todas as vezes que for tirar o leite do peito, comece lavando as mãos, com as mãos lavadas realize massagens leves no peito, com movimentos em círculo e depois de cima para baixo para ajudar o leite a sair do peito.”* (…every time you express the milk from the breast, start by washing your hands, with the washed hands perform light massages on the breast, with movements in a circle and then from top to bottom to help the milk come out of the breast)

After analyzing expert judges’ assessments, it was observed that the recommendations were about increasing the size of the letters used in some scenes and reformulating phrases in the narration text, with a view to making them more understandable by the target audience. Therefore, in the narration text, some terms, phrases and sentences were replaced.

## DISCUSSION

As we look forward to all the advances in technology, we find that PNB care remains a constant challenge to reduce the neonatal morbidity and mortality rate worldwide^(30)^. The construction and availability of educational technology contributed to the health education process^(31)^, since it optimizes the routine of nursing and health professionals, favoring parents, family members and caregivers to reduce doubts and insecurity in premature infant care^(5, 30)^.

To construct and validate the storyboard of an educational video entitled “*O cuidado do premature*” about PNB care at home, care was taken to make it accessible and adequate for the target audience, seeking and organizing, through national and international literature, current information relevant to the subject in a simple, clear and objective way. The elaboration of quality educational technologies, based on scientific evidence, makes it possible to carry out educational practices anchored in structured knowledge and directed to the target audience^(32)^. As in this study, others also appropriated national and international scientific evidence to elaborate the educational material content which they proposed to carry out^(5, 32, 33, 34)^.

The audiovisual media story-board organization, in the Activities of Daily Living Model thematic axes^(23, 24)^, made it possible to better configure the content, with accessible and relevant characteristics that are easy to understand, with applicability to practice, in addition to individualizing nursing care. It is noteworthy that, when constructing educational technology in health, the theoretical basis is necessary, as it enhances the achievement of the expected educational objective^(35, 36)^.

Storyboard content is distributed with PNB’s main daily activities, such as feeding, bathing, changing diapers, personal hygiene, controlling body temperature, administering medications, using the kangaroo position, following the appointment calendar and taking care of the main intercurrences with PNB, the most prevalent and addressed in it being choking, regurgitation and hypothermia. It is composed of 44 scenes, lasting ten minutes, in graphic animation format, with five characters that are presented throughout the video, according to the resources used, such as the presence of an announcer (on or off ) and with audiovisual media that gave dynamism to the educational technology^(37)^.

Researchers recommend that the duration of this type of audiovisual media should not exceed 15 minutes^(38, 39)^. In another study, the duration of approximately 10 minutes manages to keep the viewer’s attention more easily^(37)^.

In order to make the educational video content didactic, its construction contemplated the CTML’s 12 characteristics/uniqueness: coherence (challenging unnecessary images, words and sounds); signage (presentation of signs that direct attention); redundancy (application of animation and narration instead of animation, narration and subtitles); spatial contiguity (spatially close corresponding words and images); temporal contiguity (words and corresponding images appear at the same time); segmentation (information in chunks); pre-training (general presentation of content prior to details); modality (animation and narration rather than animation and written text); multimedia (use of words and pictures rather than just words); personalization (words in conversational rather than formal style); voice (human voice narration); image (the image of the narrator is not essential)^(16)^.

In this study, the choice of audiovisual media as a complementary strategy for guidance on home care with PNB occurred because it is a resource that is easy to access and use and has a motivating character regarding the public that it intends to target^(18, 19)^. To this end, through this graphic animation resource, the public is provided with a virtual environment with multisensory experiences and simpler, easier to understand and more effective learning^(5, 18, 19, 39)^.

It should be noted that, due to the limited number of instruments developed specifically for the neonatal population, professionals often adapt materials developed for the adult population or do not use instruments capable of demarcating or quantifying data in neonatology, thus making it important to develop and validate screening instruments, specific for this population, that are reliable, accurate, easy to apply and that do not put patients at risk^(15, 40)^.

Although the video storyboard content was extracted from recognized scientific material and thematic reference, it was necessary to validate it, in order to obtain scientific support for its content. The educational technology built was validated by 14 (fourteen) nurse judges. According to Galindo-Neto et al.^(19)^, it is necessary to choose professionals with expertise in the area of interest for content assessment, as they contribute to the material containing clear, correct, objective information, but with necessary highlights to ensure content clarity^(32, 33)^.

The recommendations suggested by judges allowed the final adjustment of three items assessed by the instrument^(26)^, being related to requirements appropriate language for the target audience, appropriate language for the educational material and logical sequence of ideas. The suggested modifications were about reformulating excerpts from the video narration and increasing the size of some of the images. These changes were also performed by other studies^(3, 10, 11, 41)^, aiming at a better understanding by the public that will use the audiovisual media content.

According to expert judges’ analysis, it was possible to make content clearer, simpler and more objective. It also made it possible to produce a more colloquial, understandable and culturally appropriate language for parents, family members and caregivers of PNB. Thus, these findings confirm the relevance of technologies aimed at health education being directed to the popular context of the target audience^(11, 42)^.

Validity by judges points to rigor in content assessment, being considered adequate for the teaching-learning process, with an overall CVI of 0.901, being identified in the construction and validity process of other educational technologies for NB care^(1, 5, 13, 43, 44)^.

### Study limitations

The limitation of this study is related to the video validity process restricted to content validity. The most varied challenges imposed by the COVID-19 pandemic for developing this study did not allow us to advance in the psychometric validity of video appearance and external validity; however, this stage will be carried out in a later study, together with the educational video effectiveness analysis with the target audience.

### Contributions to nursing, health or public health

The storyboard of “*O cuidado do prematuro*” was built with a view to improving care with PNB, expand access to the best evidence and foster the autonomy of families and caregivers of PNB with a focus on difficulties and problems compatible with reality. Its construction and validity will contribute to scientific advancement in the neonatal area, considering that the availability of this educational technology will enable the dissemination of care information and its use during health promotion actions.

The relevance of this study is given the lack of video-type educational technologies necessary for PNB caregivers, about home care, considering that PNB has vulnerabilities and singularities that need to be recognized. Moreover, this technology can collaborate with the consolidation of evidence-based practices of health professionals and with the development of competence for home care, based on the teaching-learning process. There is the possibility that the educational video can be used as a pedagogical strategy by both undergraduate nursing professors and nurses to streamline and innovate health education approaches about PNB care at home and to complement the guidelines given in the hospital discharge process, home visits or group approaches.

## CONCLUSIONS

The storyboard of an audiovisual media entitled “*O cuidado do prematuro*” was built from graphic animation, being validated in terms of content by expert judges in neonatal care. It lasts ten minutes and contemplates the conceptual structures of the nursing theory of activities of daily living, to meet needs regarding PNB care. The video storyboard also included the principles of CTML. The educational video content was considered valid in terms of objectives, structure, presentation, relevance and content, based on CVI analysis, which showed values equal to or greater than 80%, given that those that did not receive experts’ suggestions were taken care of and corrected in the storyboard.

The need to validate new educational technologies is pointed out, including other experts, not only in the nursing area, to expand the interdisciplinary nature of PNB care. It is hoped that this video can contribute to constructing knowledge about PNB care, streamline and innovate health education approaches as well as facilitate the clarification of doubts, transforming learning into attractive situations and the capacity for critical-reflective analysis of people who watch the educational video.
